# Psychiatric comorbidities in Asperger syndrome are related with polygenic overlap and differ from other Autism subtypes

**DOI:** 10.1038/s41398-020-00939-7

**Published:** 2020-07-30

**Authors:** Javier González-Peñas, Javier Costas Costas, Alicia García-Alcón, María José Penzol, Julio Rodríguez, Cristina Rodríguez-Fontenla, Aitana Alonso-González, Montse Fernández-Prieto, Ángel Carracedo, Celso Arango, Mara Parellada

**Affiliations:** 1 Instituto de Investigación Sanitaria Gregorio Marañón (IiSGM), Centro de Investigación Biomédica en Red en Salud Mental (CIBERSAM), Madrid, Spain; 2 Grupo de Medicina Xenómica, Fundación Instituto de Investigación Sanitaria de Santiago de Compostela (FIDIS), Santiago de Compostela, Spain; 3 Instituto de Investigación Sanitaria Gregorio Marañón (IiSGM), Madrid, Spain; 4 Grupo de Medicina Xenómica, CIBERER, CIMUS (Centre for Research in Molecular Medicine and Chronic Diseases), Universidade de Santiago de Compostela, Santiago de Compostela, Spain; 5 Grupo de Medicina Xenómica, Fundación Instituto de Investigación Sanitaria de Santiago de Compostela (FIDIS), CIBERER, CIMUS (Centre for Research in Molecular Medicine and Chronic Diseases), Universidade de Santiago de Compostela, Santiago de Compostela, Spain; 6 Instituto de Investigación Sanitaria Gregorio Marañón (IiSGM), Centro de Investigación Biomédica en Red en Salud Mental (CIBERSAM), Facultad de Medicina, Universidad Complutense, Madrid, Spain

**Keywords:** Personalized medicine, Autism spectrum disorders

## Abstract

There is great phenotypic heterogeneity within autism spectrum disorders (ASD), which has led to question their classification into a single diagnostic category. The study of the common genetic variation in ASD has suggested a greater contribution of other psychiatric conditions in Asperger syndrome (AS) than in the rest of the DSM-IV ASD subtypes (Non_AS). Here, using available genetic data from previously performed genome-wide association studies (GWAS), we aimed to study the genetic overlap between five of the most related disorders (schizophrenia (SCZ), major depression disorder (MDD), attention deficit hyperactivity disorder (ADHD), obsessive-compulsive disorders (OCD) and anxiety (ANX)), and AS, comparing it with the overlap in Non_AS subtypes. A Spanish cohort of autism trios (*N* = 371) was exome sequenced as part of the Autism Sequencing Consortium (ASC) and 241 trios were extensively characterized to be diagnosed with AS following DSM-IV and Gillberg’s criteria (*N* = 39) or not (*N* = 202). Following exome imputation, polygenic risk scores (PRS) were calculated for ASD, SCZ, ADHD, MDD, ANX, and OCD (from available summary data from Psychiatric Genomic Consortium (PGC) repository) in the Spanish trios’ cohort. By using polygenic transmission disequilibrium test (pTDT), we reported that risk for SCZ (*P*_scz _= 0.008, corrected-*P*_SCZ_ = 0.0409), ADHD (*P*_ADHD_ = 0.021, corrected-P_ADHD_ = 0.0301), and MDD (*P*_MDD_ = 0.039, corrected-P_MDD_ = 0.0501) is over-transmitted to children with AS but not to Non_AS. Indeed, agnostic clustering procedure with deviation values from pTDT tests suggested two differentiated clusters of subjects, one of which is significantly enriched in AS (*P* = 0.025). Subsequent analysis with S-Predixcan, a recently developed software to predict gene expression from genotype data, revealed a clear pattern of correlation between cortical gene expression in ADHD and AS (*P* < 0.001) and a similar strong correlation pattern between MDD and AS, but also extendable to another non-brain tissue such as lung (*P* < 0.001). Altogether, these results support the idea of AS being qualitatively distinct from Non_AS autism and consistently evidence the genetic overlap between AS and ADHD, MDD, or SCZ.

## Introduction

Autism spectrum disorders (ASD) comprise a group of complex neurodevelopmental disorders characterized by restricted interests, impaired social interaction, and stereotyped and repetitive behaviors^1^. Epidemiological studies reflect that around 1% of worldwide population could have ASD diagnosis^2^. The lack of empirical evidence to support the appropriateness of different ASD subtypes has led to their classification into one single category. Part of the clinical heterogeneity of ASD has to do with the coexistence with psychiatric and medical conditions^3–9^. Differential psychiatric comorbidities may have an etiopathological relationship with the ASD subtype, as previously conceptualized.

Major depression (MDD), anxiety (ANX), attention deficit hyperactivity disorder (ADHD), schizophrenia (SCZ), and obsessive-compulsive disorders (OCD) are among the most prevalent psychiatric comorbidities in ASD patients. This clinical overlap has been more extensively studied in high functioning autism (HFA) and in Asperger syndrome (AS), which may be biasing the findings^10–14^.

Recent advances in ASD genomics have demonstrated a clear pattern of biological overlap between ASD and other psychiatric conditions^14–18^. While ASD predisposing rare variation shows a clear overlap with severe neurodevelopmental disorders, intellectual disability and, in lesser degree, schizophrenia^16^, is the common variation that seems to be shared across major psychiatric disorders^14,15^. For instance, strong genetic correlations between ASD and MDD (*r*_G_ = 0.412; *P* < 10^−24^), SCZ (*r*_G_ = 0.212; *P* < 10^−24^) or ADHD (*r*_G_ = 0.360; *P* < 10^−11^) have been described in terms of common variation^15^. In this sense, the latest ASD GWAS to date demonstrated that common variation contribution to Asperger (AS) subtype was almost double the contribution to other DSM-IV ASD subtypes^15^. Given this main difference in the genetic architecture of AS, a reasonable question is whether common predisposing variation from comorbid disorders may be present to a greater extent in AS than in other ASD subtypes.

In addition, the criteria for an appropriate AS diagnosis is, still, a matter of important debate^19–22^. In a previous study, our group surveyed subclinical psychopathology in children and adolescents diagnosed with AS following DSM-IV and Gillberg’s criteria, describing high sub-syndromic MDD, OCD, ADHD symptomatology in our AS sample^23^.

Therefore, in this study, we aimed to assess whether polygenic risk for ASD and comorbid disorders are differently transmitted in AS compared with other autism subtypes (Non_AS). Using polygenic transmission disequilibrium test, a method previously performed in a larger ASD cohort^18^, we analyzed polygenic transmission using an ASD cohort of 379 trios and tested whether comorbid disorders were over-transmitted in AS but not in Non_AS trios. We then used hierarchical clustering to classify subjects based on their transmitted polygenic scores. Indeed, to more deeply understand the significance of this polygenic contribution, we used recent developed *software*, PrediXcan^24^ and S-PrediXcan^25^ to analyze how over-transmitted alleles in AS subjects affect brain cortical gene expression.

## Methods

### ASD sample for polygenic score calculation (target sample)

ASD complete trios (*N* = 379) were recruited in two sites in Spain, Santiago de Compostela (*N* = 138; sample 1), and Madrid (*N* = 241; sample 2). Subjects from Madrid were recruited as part of AMITEA program, at the Department of Child and Adolescent Psychiatry, Hospital General Universitario Gregorio Marañón. Subjects from Santiago were recruited from an ongoing project from the Galician Public Foundation of Genomic Medicine. Informed consent signed by each participating subject or legal guardian and approval from the corresponding Research Ethics Committee were obtained before starting the study. Only individuals with 3 years of age or above and ASD diagnosis were included. All trios recruited were complete. Parents had no diagnosed psychiatric disorder when trios were recruited.

Diagnosis of ASD for sample 2 was done by Child Psychiatrists with extensive experience in ASD, clinically trained in the autism diagnostic interview-revised (ADI-R) and research-trained in the autism diagnostic observation schedule (ADOS). All diagnoses followed Diagnostic and Statistical Manual of Mental Disorders, Fourth Edition Text Revision and Fifth Edition (DSM-IV-TR and DSM-5) criteria^26^. When necessary (due to inconsistencies found between all the information available), the ADOS and/or ADI-R were also administered. For the comorbidity study, we used only participants from the Madrid site, with 39 trios with this specific AS diagnosis, and 202 with other ASD diagnoses (Non_AS), since only these 241 trios were phenotyped enough to ensure a possible diagnosis of AS. These subjects were diagnosed with AS if they had an ASD without mental retardation diagnosis and met Gilbert’s criteria for AS^19^. In fact, 41 probands without mental retardation were included within the 202 Non_AS trios, as they did not meet both DSM and Gilbert’s criteria. No screening for copy number variants (CNVs) carriers was performed. The average age for recruitment and diagnoses was 15.41 years (CI 95%: 14.55–16.66).

### Polygenic scores calculation

We calculated polygenic risk scores (PRS) in the 379 Spanish ASD trios that were sequenced as part of the Autism Sequencing Consortium (ASC). Firstly, exome-based VCF files were imputed at Michigan Imputation Server (https://imputationserver.sph.umich.edu/) using 1000 Genome Project phase 3v.5 reference panel in order to capture genomic variants beyond exome. From 654,286 variants, a total of 38.7 million were obtained. Only biallelic variants with imputation quality score > 0.9 and minor allele frequency (MAF) > 0.1% were considered (72,292 genotyped and 997,210 imputed SNPs were retained). Then, exome-based PRS scores were calculated. Briefly, GWAS *summary statistics* from PGC repository (https://www.med.unc.edu/pgc/results-and-downloads) were used as discovery sample (Supplementary Table 1). PRS was assigned to each individual from target sample as the sum of number of effect alleles weighted by their effect in the discovery sample. Indels were also excluded. Clumping was performed using PLINK v1.9 code *“--clump-r2 0.1 --clump-kb 500*”. We used *Flip Strand* and remove ambiguous genomic positions. For each individual, we calculated PRS scores for ASD^15^ and for five comorbid disorders here surveyed: MDD^27^, SCZ^28^, ADHD^29^, ANX^30^, and OCD^31^. We generated different PRS using various *P* thresholds (*P* < 0.01, 0.05, 0.1, 0.2, 0.5, and 1) in the discovery dataset for inclusion of SNPs below these thresholds in PRS and calculated polygenic transmission disequilibrium test (pTDT), using parent-child trios PRS information, as previously performed in ASD^18^. Briefly, the expected PRS distribution of the offspring is compared with the average PRS distribution of the parents, and its deviations is tested with a one-sample *t*-test. For each disorder, the *P* threshold with the highest estimate value of transmission from pTDT on the whole sample (sample 1 and sample 2) was selected and henceforth used for comparison between AS (*N* = 39) and Non_AS (*N* = 202 trios; sample 2) trios. As a negative control, pTDT analysis was repeated using summary statistics of body mass index (BMI), from recent meta-analysis^32^, a trait without genetic correlation with ASD.

### Clustering classification based on transmitted genetic scores

In order to analyze substructure of our ASD cohort based on polygenic transmission, we performed an agnostic procedure of clustering classification (without specifying the number of clusters to be generated). We first determined the more suitable method using the R package *clValid*^33^. Briefly, *clValid* performs clustering to analyze the input data by using many different algorithms (hierarchical, self-organizing maps (SOM), *K*-means, self-organizing tree algorithm (SOTA), and model-based). The most suitable method is suggested after an internal validation procedure implemented. The internal measures include the connectivity (the degree of connectedness of the clusters), Silhouette Width (the degree of confidence in a particular clustering assignment), and Dunn Index (the ratio between the smallest distance between observations not in the same cluster to the largest intra-cluster distance). We then used *NbClust* R package^34^ to determine the optimal number of clusters making use of different indexes and varying all combinations of number of clusters, distance measures, and clustering methods.

Hierarchical clustering using Ward’s minimum variance method was performed with *hclust* function from *cluster* R package. Goodness of clustering algorithm results was assessed by determining the silhouette width coefficient, which measures how well an observation is clustered and it estimates the average distance between clusters. Observations with negative silhouette width were removed. *fviz_silhouette* function from *factoextra* R library was used. Finally, ASD subjects within each cluster were analyzed to test enrichment of AS subjects within any group.

### Gene expression prediction

PrediXcan, a recently published *software* that imputes tissue-specific gene expression levels from subject’s genetic profile^24^, was used to assess gene expression differences between patients from AS and Non_AS trios. Briefly, PrediXcan makes use of available expression quantitative trait loci (eQTLs) data from GTEx project^35^ to predict expression levels in a tissue-dependent manner and compares expression levels across different phenotypes. Genotype files from ASD trios with available diagnose information (*N* = 241) were converted from.*bed* to.*dosage* format. Predixcan was run with --*predict* and –*assoc --logistic* arguments to predict expression levels per individual and perform statistical comparisons per gene between AS and Non_AS subjects. Given the extensive literature connecting cortical dysfunctions and autism, either from imaging^36^, gene expression^37–39^ or rare disrupting variation^40^ studies, GTEx brain frontal cortex was used as reference transcriptome. Differences in predicted gene expression in frontal cortex between AS and Non_AS patients were assessed.

S-PrediXcan^25^, an extension of PrediXcan software that infers its results using only summary statistics from GWAS, was then used on GWAS summary data from ASD and comorbid disorders with significant polygenic transmission (SCZ, MDD, ADHD) to estimate gene expression differences between cases and controls using imputed expression data from GTEx (brain frontal cortex).

Correlation between gene expression differences between AS and Non_AS (measured by per gene *Z-*values derived from PrediXcan test) and gene expression differences between cases and controls from comorbid disorders (measured by per gene *Z-*values derived from S-PrediXcan test) was analyzed. This relation was studied under different *P* thresholds from S-PrediXcan.

GTEx expression data from lung and cerebellum, other brain tissue with less consistent evidences in psychiatry that frontal cortex^41^ were used as comparative controls. Body mass index (BMI) summary statistics^32^ were also used in S-PrediXcan analyses in same tissues as a negative control, as it has not been associated with ASD across literature and repeatedly used as control. Finally, Alzheimer disease (ALZ) summary statistics^42^ were used in S-PrediXcan analyses in same tissues as a comparison between neurodevelopment and a neurodegenerative disorder.

### Statistical analyses

To assess for polygenic over-transmission in AS and Non_AS, one-sided *t*-test were performed in form of pTDT. Full procedure is described in previous work^18^. Comparisons of polygenic transmission values between AS and Non_AS groups were performed with two-sample *t*-test. Data normality was contrasted with Shapiro–Wilk test. Paired *t*-test was used to test significance of transmission across all comorbid disorders in AS and Non_AS subgroups of trios. Correlation analyses were performed using Spearman correlation. In case of multiple test comparison, Benjamini–Hochberg FDR correction was performed.

## Results

### Polygenic transmission disequilibrium

After imputation and quality control (QC) of genotypic data, we retained 221,418 SNPs, of which a total of 51,718 variants with MAF > 0.1% were used after clumping (methods). Average pTDT and deviation were calculated in our ASD trios cohort using GWAS data from ASD and five comorbid disorders assessed (SCZ, MDD, ADHD, ANX, and OCD; Table 1). All subsequent analyses were based on the *P* threshold in which pTDT had higher transmission values at each disorder.

**Table 1:** Polygenic transmission estimate values (from pTDT tests) of ASD and studied comorbid disorders (ASD, SCZ, MDD, ADHD, ANX, and OCD) in all ASD trios’ population (*N* = 379).

*P* threshold	ASD	SCZ	MDD	ADHD	ANX	OCD	BMI
*P* < *0.001*	−0.022	0.053	0.066	0.018	0.029	−0.083	**0.064**
*P* < *0.01*	0.054	0.598	**0.132**	**0.061**	**0.07**	−0.047	0.027
*P* < *0.05*	0.016	0.051	0.117	0.028	−0.013	**−0.019**	0.051
*P* < *0.1*	0.02	0.03	0.058	0.049	0.005	−0.07	0.036
*P* < *0.2*	0.034	**0.063**	0.022	0.033	−0.035	−0.047	0.039
*P* < *0.5*	**0.056**	0.041	0.03	0.047	−0.049	−0.025	0.039
*P* < *1*	0.054	0.041	0.055	0.035	−0.042	−0.023	0.04

Several *P* thresholds (from GWAS summary statistics) were used in order to select the optimal cutoff for PRS calculation. highest transmission value from pTDT test in each case is marked in bold.

**Fig. 1:**
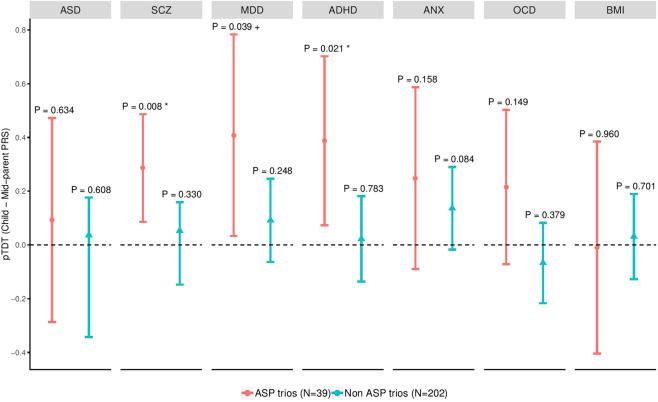
Polygenic transmission of ASD, comorbid disorders and BMI AS trios (*N* = 39), and Non_AS trios (*N* = 202). Transmission disequilibrium is represented as standard deviations of the mid-parent distribution. Colored geometric lines represent 95% confidence intervals. *P-*values over geom error bars measure the probability that the mean of the pTDT deviation distribution is higher than 0 (two-sided, one-sample *t-*test). For each disorder, pTDT values were calculated based on the *P* threshold in which highest transmission were found for the whole ASD trios’ population (*N* = 379). Significant over-transmissions were confirmed with random permutation of AS subgroup. “*” Permutation *P-*value < 0.05; “+” Permutation *P*-value < 0.1.

ASD trios with available full diagnosis (*N* = 241) were used to compare pTDT values between AS and Non_AS. Polygenic risk for SCZ, ADHD and MDD was significantly over-transmitted to AS affected children in trios (*P* < 0.05; Fig. 1), but not to Non_AS affected children (*P* > 0.05). We confirmed these results by conducting 1000 random permutations of diagnosis status (permutation-*P*_SCZ_ = 0.0409; permutation-*P*_MDD_ = 0.051; permutation-*P*_ADHD_ = 0.0301). As a negative control, pTDT analysis was repeated using BMI summary statistics. Polygenic over-transmission was neither found in AS nor in Non_AS subgroup (*P* > 0.05 for both comparisons; Supplementary Table 2). Although no significance was found in ASD, OCD, or ANX polygenic risks in the AS subgroup of patients, we observed higher polygenic transmission in the AS group for all psychiatric-related disorders assessed in this study. This pattern of over-transmission of psychiatric-related polygenic risks is, in fact, unlikely to happen by chance (Paired *t*-test *P* = 0.02175, CI (95%) = 0.04562–0.37237).

Correlation of pTDT deviations across disorders was assessed (Supplementary Fig. 1). Although SCZ and OCD pTDT deviations are significantly correlated within our ASD cohort, there are no correlation between any of significantly over-transmitted pTDT scores (ADHD, SCZ, and MDD). Therefore, multiple polygenic over-transmission does not occur due to genetic overlap between disorders. Instead, different genetic etiologies contribute to the development of AS condition is a more likely outcome.

We finally performed clustering using all psychiatric pTDT deviation values from AS and Non_AS trios. Hierarchical clustering was determined as the most suitable method (Supplementary Table 3). Two different clusters (C1 and C2) were observed with the highest probability (Supplementary Table 4; Supplementary Fig. 2; 165 and 76 subjects in C1 and C2, respectively). Individuals with negative silhouette classification values were removed (Supplementary Fig. 3), pruning clusters with only confident results (118 and 74 individuals in C1 and C2, respectively). AS subjects were overrepresented in cluster C2 (18 of 74 vs. 13 of 118; Fisher two-tailed *P* = 0.025; OR (95%) = 2.60 (1.10–6.19)).

### Prediction of brain frontal cortex gene expression

In order to analyze whether polygenic over-transmission related with comorbid disorders (MDD, ADHD, and SCZ) in AS subgroup has biological consequences, we used two recent *software*, Predixcan and S-PrediXcan (methods), to infer gene expression differences between AS and Non_AS, and to compare these differences with expression patterns related with those comorbid disorders.

We used gene expression predictions from brain frontal cortex, a tissue extensively related with ASD. Gene expression was predicted from our imputed exome data. Two-hundred twenty-six out of 4111 imputable genes were differently expressed (*P* < 0.05) in AS vs. Non_AS subjects. However, no gene reached statistical significance in predicted gene expression differences after correcting for multiple testing (Supplementary Table 5).

**Fig. 2:**
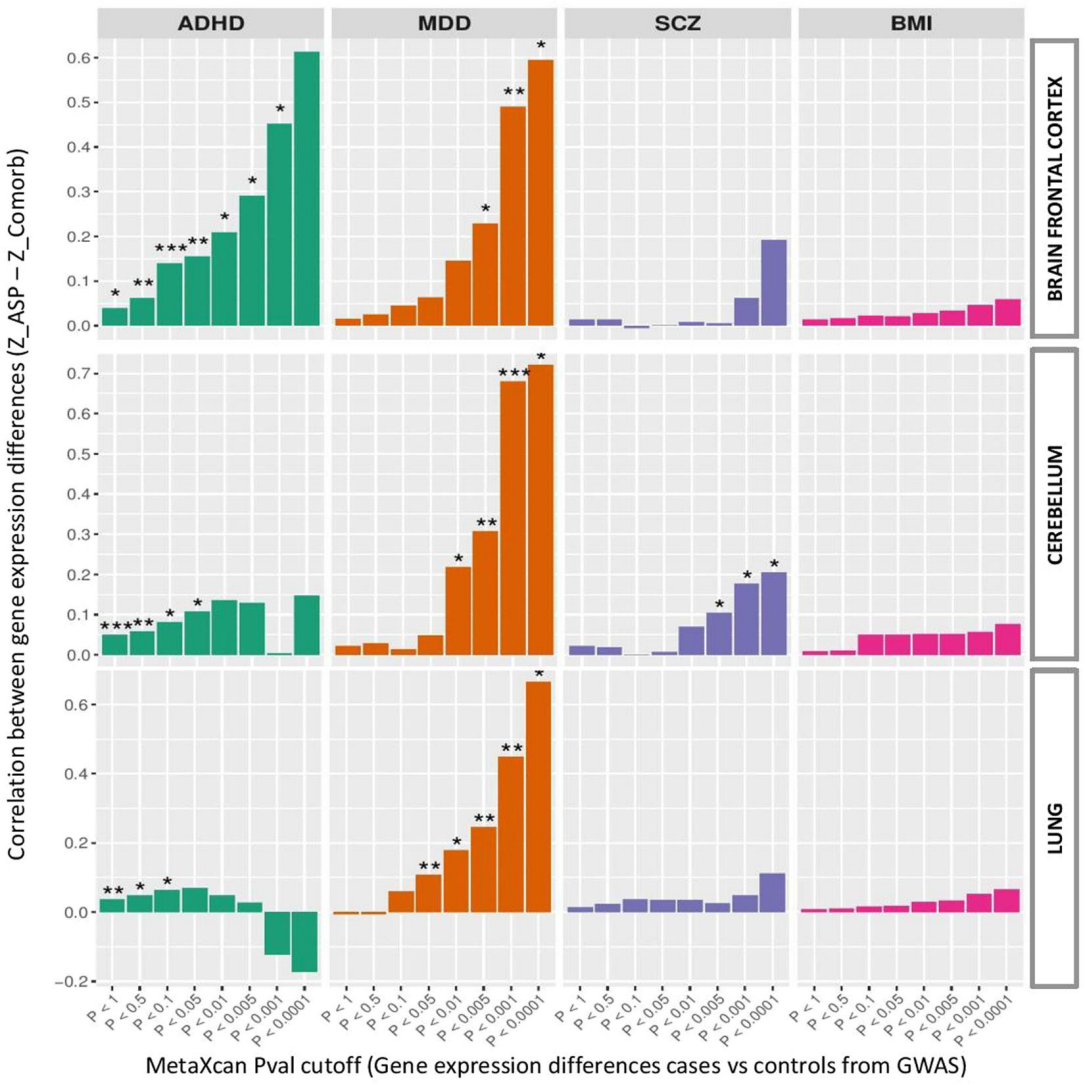
Spearman correlation between gene expression diferences in AS against Non_AS and gene expression differences in comorbid disorder (ADHD, MDD, and SCZ). Correlation under various *P* cutoffs from S-PrediXcan results (imputed gene expression differences in cases vs. controls from ADHD, MDD, SCZ, and BMI GWAS), were assessed. Predicted expression relationships were studied in brain frontal cortex, cerebellum, and lung. BMI was used as a negative control disorder. *P < 0.05; **P < 0.01; ***P < 0.001. Gene numbers and correlation results are described in Supplementary Table 6.

*Z-*values from PrediXcan test, regarding gene expression differences in brain frontal cortex between AS and non AS patients, and *Z-*values from S-PrediXcan test, regarding gene expression differences in cases and controls for each comorbid disorder, were compared to study whether cortical gene expression profiles in AS compared to Non_AS resemble gene expression profiles in comorbid disorders with significant polygenic transmission in AS trios (ADHD, MDD, and SCZ; Fig. 2 and Supplementary Table 6). We observed a significant pattern of correlation between ADHD and AS gene expression patterns in brain frontal cortex (Fig. 2; most significant Spearman Corr = 0.135; *P* = 8.832 × 10^−4^ (*P*_threshold _= 0.1)). When restricting analysis to genes more differently expressed from S-PrediXcan results (ADHD vs. controls), correlation systematically grows, indicating that the more specific ADHD genes in terms of predicted gene expression, the higher correlation with gene expression differences between AS and Non_AS samples. Interestingly, this growing correlation pattern was neither reproducible in cerebellum nor in lung, reflecting that polygenic overlap between ADHD and AS may have high impact in brain cortex but not in other tissues (Fig. 2).

We also identified significant correlation between MDD and AS gene expression patterns in brain frontal cortex (Fig. 2; most significant Spearman Corr = 0.490; *P* = 7.65 × 10^−3^ (*P*_threshold _= 0.001)), and similar growing patterns of correlation were observed when restricting genes analyzed to those more differentially expressed from S-PrediXcan results (lowering *P*_threshold_). However, when comparing expression differences across other tissues, significant correlation and same growing correlation trends were observed in cerebellum (Fig. 2; most significant Spearman Corr = 0.68; *P* = 1.64 × 10^−5^ (*P*_threshold_ = 0.001)) and lung (Fig. 2; most significant Spearman Corr = 0.44; *P* = 4.49 × 10^−3^ (*P*_threshold_ = 0.001)). Thus, expression similarities between MDD and AS conditions appear not to be restricted to brain cortex but distributed across other tissues.

Contrary to MDD and ADHD, polygenic overlap between AS and SCZ could not be explained in terms of predicted expression differences in brain frontal cortex, where no significant correlations were found (Supplementary Table 6). Significant but less consistent correlations were found in cerebellum (Fig. 2; most significant Spearman Corr = 0.176; *P* = 0.016 (*P*_threshold _= 0.001)), but to a significant lesser degree than findings in ADHD or MDD.

To assess whether these correlations patterns are extendable to a neurodegenerative disorder, AS and ALZ gene expression correlation was also explored. In contrast with ADHD or MDD, no consistent growing pattern of correlation was observed (Supplementary Table 6), which suggests that expression similarities between AS and neuropsychiatric disorders are restricted to neurodevelopment.

Indeed, no correlation pattern was observed when using BMI results from S-PrediXcan in any of the three tissues analyzed (Fig. 2).

## Discussion

The results presented here confirm that the ASD polygenic contribution is shared with other psychiatric disorders, such as SZ, MDD, and ADHD, but restricted to AS (DSM-IV plus Gillberg criteria) instead of the whole ASD sample. Indeed, for the five described comorbid disorders studied here (SCZ, MDD, ADHD, ANX, and OCD), the polygenic contribution is higher in AS subtype of ASD than in Non_AS autism (Fig. 1). This higher contribution of common variation in AS has been recently described in the largest ASD GWAS performed to date^15^. Although diagnostic criteria used in both studies vary slightly, the results presented here are consistent with those from Grove et al. Insofar, a higher contribution from common variation arises in HFA compared to syndromic forms or other subtypes of autism with marked intellectual affectation, who seem to be more affected by rare disruptive variation^40^. The differential genetic contributions in different clinical subtypes of ASD could partially explain the weaker correlations observed between ASD and other psychiatric disorders in previous cross-disorder studies based on both genomic^14^ or transcriptomic^43^ data.

A main utility of PRS is the possibility to evaluate the genetic contributions of different disorders on well-characterized subtypes of a target sample under study^15,44–46^. In this study, by performing pTDT^18^, which uses family-based samples, results are not affected by ancestral stratification or other environmental factors that potentially biases case–control studies, such as socioeconomic status.

Given that no correlation between ASD and comorbid disorders (SZ, MDD, and ADHD) was observed at the pTDT level (Supplementary Fig. 1), we performed hierarchical clustering using pTDT values and observed two main clusters with one of them enriched in AS subtype (*P* = 0.025; Supplementary Fig. 2). Thus, by using an agnostic procedure of hierarchical clustering we strengthen the rationale of our initial hypothesis.

Whole-genotype data was used to predict gene expression differences between AS and Non_AS subjects^24^. Similarly, GWAS summary statistics were used to infer gene expression differences between cases and controls from studied comorbid disorders^25^. By comparing AS–Non_AS and case–control differences from comorbid disorders, we described significant gene expression relationships between AS and ADHD or MDD.

These results suggest that the polygenic risk shared between AS and other psychiatric disorders is mediated mainly through changes in brain gene expression. In this sense, while we show that expression correlations between AS and ADHD are restricted to prefrontal brain cortex, we observe gene expression correlations between AS and MDD extending to cerebellum and beyond brain tissue, with notorious correlation even in lung tissue, suggesting involvement of genes with broad expression related to general biological processes. Although these expression analyses from PrediXcan^24^ and S-PrediXcan^25^ software rely on imputed data using eQTL information from GTEx database^35^, they have been already extensively used in psychiatric genetics as a reliable approach to infer tissue-specific gene expression values^47,48^.

One of the greatest novelties of our study is the combination of DSM-IV and Gillberg criteria to diagnose AS subtype. This coincides with Klin et al.^20^ proposal of giving precedence to the diagnosis of AS when both AS and Autism criteria are met. Unique features of AS such as social motivation, awkward one-sided social approaches or precocious language, among others^20,49^, are considered when clinically diagnosing AS as a subtype separated from HFA. Lack of empirical evidence for distinct subtypes derived in lumping all subtypes of ASD within a same category in the DSM-5^50^. Apart from brain connectivity differences^51–54^, very little neurobiological data support the distinction between AS and HFA. The qualitative differences between AS and HFA with respect to psychiatric comorbidity have been hitherto explained by psychological mechanisms^50^. Thus, having social motivation but being incompetent in appropriately approaching others might lead to greater difficulties in social adjustment than being more uninterested in social relationships (as in the aloof subtype described by Lorna Wing^22^), which may lead to greater comorbidity with depressive disorders, particularly in adolescence and the transition to adult life. However, the debate whether these features characterize a subtype within HFA or personality traits is beyond the scope of this article. Data presented here are consistent with previous clinical studies^23^, and we extract from our results that AS meeting Gillberg’s criteria might be a highly polygenic disorder and strongly affected by other psychiatric contributions.

However, our observations do contrast with those from other recent studies^15^, and place AS, in terms of genetic contributions, closer to conditions as MDD or ADHD rather than to average ASD. From a nosological perspective these results may add to the discussion whether genetic contribution may help subtyping the group of disorders comprised in the term ASD^55^.

We have to be aware of important limitations in our study, including the small sample size of the sample and its limited phenotypic characterization. Intriguingly, we did not observe significant polygenic over-transmission of ASD common variation in either AS or Non_AS subtypes (Fig. 1). There are two main reasons that may be behind this counterintuitive result. Firstly, a bigger sample size could be necessary to increase the power of the analyses and reduce the pTDT confidence intervals’ width. Secondly, PRS prediction power in target sample strongly depends on discovery sample size, which varies among the disorders here tested (for instance: *N*_ASD_GWAS_ = 46,351, whereas *N*_MDD_GWAS_ = 480,359). Moreover, other power dependencies as phenotypic heterogeneity of ASD discovery sample might also explain these results. In this sense, similar inconsistencies have been previously found^56^, pointing to ASD clinical heterogeneity as a big obstacle for explaining the expected variance in PRS analyses. A deeper phenotypic characterization of the sample (particularly within ASD without intellectual disability) could help better understanding which patients may have higher polygenic risk for comorbid conditions. Indeed, we may not discard the possibility that some forms of high functioning autism, i.e., AS meeting Gillberg criteria, or any HFA with social motivation, could have stronger polygenic psychopathological contributions^57^ than others.

The results herein presented of a polygenic overlap and correlated gene expression profiles between AS and other psychiatric disorders support the idea of AS being qualitatively distinct from Non_AS autism. This adds to previous evidence showing high psychopathological comorbidity (at the symptom level) between AS and SCZ, ADHD, and MDD^23^. The data presented here, particularly that related to comorbidity with ADHD, adds on previous elucubrations giving the possibility of a more biological-based explanation of the high psychiatric comorbidity observed in AS.

## Supplementary information

Supplementary Information

Supplementary Information

## References

[1] Bourgeron T (2015). From the genetic architecture to synaptic plasticity in autism spectrum disorder. Nat. Rev. Neurosci..

[2] Baxter AJ (2015). The epidemiology and global burden of autism spectrum disorders. Psychol. Med..

[3] Lord C, Jones RM (2012). Annual research review: re-thinking the classification of autism spectrum disorders. J. Child Psychol. Psychiatry.

[4] Wing L, Gould J, Gillberg C (2011). Autism spectrum disorders in the DSM-V: better or worse than the DSM-IV. Res. Dev. Disabil..

[5] Joshi G (2013). Psychiatric comorbidity and functioning in a clinically referred population of adults with autism spectrum disorders: a comparative study. J. Autism Dev. Disord..

[6] Lever AG, Geurts HM (2016). Psychiatric co-occurring symptoms and disorders in young, middle-aged, and older adults with autism spectrum disorder. J. Autism Dev. Disord..

[7] Maddox BB, White SW (2015). Comorbid social anxiety disorder in adults with autism spectrum disorder. J. Autism Dev. Disord..

[8] Reiersen AM, Constantino JN, Volk HE, Todd RD (2007). Autistic traits in a population-based ADHD twin sample. J. Child Psychol. Psychiatry.

[9] Vannucchi G (2014). Clinical features, developmental course, and psychiatric comorbidity of adult autism spectrum disorders. CNS Spectr..

[10] Lugnegård T, Hallerbäck MU, Gillberg C (2011). Psychiatric comorbidity in young adults with a clinical diagnosis of Asperger syndrome. Res. Dev. Disabil..

[11] Matson JL, Cervantes PE (2014). Commonly studied comorbid psychopathologies among persons with autism spectrum disorder. Res. Dev. Disabil..

[12] Mukaddes NM, Hergüner S, Tanidir C (2010). Psychiatric disorders in individuals with high-functioning autism and Asperger’s disorder: similarities and differences. World J. Biol. Psychiatry.

[13] Russell AJ, Mataix-Cols D, Anson M, Murphy DG (2005). Obsessions and compulsions in Asperger syndrome and high-functioning autism. Br. J. Psychiatry.

[14] Cross-Disorder Group of the Psychiatric Genomics Consortium. Identification of risk loci with shared effects on five major psychiatric disorders: a genome-wide analysis. *Lancet* **381**, 1371–1379 (2013). 10.1016/S0140-6736(12)62129-1 PMC3714010 23453885

[15] Grove J (2019). Identification of common genetic risk variants for autism spectrum disorder. Nat. Genet..

[16] Sanders SJ (2015). Insights into Autism spectrum disorder genomic architecture and biology from 71 risk loci. Neuron.

[17] Anney RJL (2017). Meta-analysis of GWAS of over 16,000 individuals with autism spectrum disorder highlights a novel locus at 10q24.32 and a significant overlap with schizophrenia. Mol. Autism.

[18] Weiner DJ (2017). Polygenic transmission disequilibrium confirms that common and rare variation act additively to create risk for autism spectrum disorders. Nat. Genet..

[19] Gillberg C (1998). Asperger syndrome and high-functioning autism. Br. J. Psychiatry.

[20] Klin A, Pauls D, Schultz R, Volkmar F (2005). Three diagnostic approaches to Asperger syndrome: implications for research. J. Autism Dev. Disord..

[21] Kopra K, von Wendt L, Nieminen-von Wendt T, Paavonen EJ (2008). Comparison of diagnostic methods for asperger syndrome. J. Autism Dev. Disord..

[22] Wing L (1981). Asperger’s syndrome: a clinical account. Psychol. Med..

[23] Caamaño M (2013). Psychopathology in children and adolescents with ASD without mental retardation. J. Autism Dev. Disord..

[24] Gamazon ER (2015). A gene-based association method for mapping traits using reference transcriptome data. Nat. Genet..

[25] Barbeira AN (2018). Exploring the phenotypic consequences of tissue specific gene expression variation inferred from GWAS summary statistics. Nat. Commun..

[26] American Psychiatric Association. Diagnostic and Statistical Manual of Mental Disorders (DSM-5®) (American Psychiatric Pub, 2013).

[27] Wray NR (2018). Genome-wide association analyses identify 44 risk variants and refine the ge.netic architecture of major depression. Nat. Genet..

[28] Ripke S (2014). Biological insights from 108 schizophrenia-associated genetic loci. Nature.

[29] Demontis D (2019). Discovery of the first genome-wide significant risk loci for attention deficit/hyperactivity disorder. Nat. Genet..

[30] Otowa T (2016). Meta-analysis of genome-wide association studies of anxiety disorders. Mol. Psychiatry.

[31] Arnold PD (2018). Revealing the complex genetic architecture of obsessive-compulsive disorder using meta-analysis. Mol. Psychiatry.

[32] Yengo L (2018). Meta-analysis of genome-wide association studies for height and body mass index in ∼700000 individuals of European ancestry. Hum. Mol. Genet..

[33] Brock, G., Pihur, V., Datta, S. & Datta, S. *clValid: An R Package for Cluster Validation. R Package Version 0.6-6*. https://CRAN.R-project.org/package=clValid (2018).

[34] Charrad, M., Ghazzali, N., Boiteau, V. & Niknafs, A. *Package NbClust. R package version 3.0*. https://cran.r-project.org/web/packages/NbClust/index.html (2015).

[35] Lonsdale J (2013). The Genotype-Tissue Expression (GTEx) project. Nat. Genet..

[36] van Rooij D (2018). Cortical and subcortical brain morphometry differences between patients with autism spectrum disorder and healthy individuals across the lifespan: results from the ENIGMA ASD Working Group. Am. J. Psychiatry.

[37] Parikshak NN (2016). Genome-wide changes in lncRNA, splicing, and regional gene expression patterns in autism. Nature.

[38] Stoner R (2014). Patches of disorganization in the neocortex of children with autism. N. Engl. J. Med..

[39] Willsey AJ (2013). Coexpression networks implicate human midfetal deep cortical projection neurons in the pathogenesis of autism. Cell.

[40] Li, J. et al. A comparative study of the genetic components of three subcategories of autism spectrum disorder. *Mol. Psychiatry*. [published online: June 06, 2018]. 10.1038/s41380-018-0081-x. 10.1038/s41380-018-0081-x 29875476

[41] Phillips JR, Hewedi DH, Eissa AM, Moustafa AA (2015). The cerebellum and psychiatric disorders. Front. Public Health.

[42] Jansen IE (2019). Genome-wide meta-analysis identifies new loci and functional pathways influencing Alzheimer’s disease risk. Nat. Genet..

[43] Anttila V (2018). Analysis of shared heritability in common disorders of the brain. Science.

[44] Costas J (2016). Exon-focused genome-wide association study of obsessive-compulsive disorder and shared polygenic risk with schizophrenia. Transl. Psychiatry.

[45] Ruderfer DM (2018). Genomic Dissection of Bipolar Disorder and Schizophrenia, Including 28 Subphenotypes. Cell.

[46] Markota, M. et al. Association of schizophrenia polygenic risk score with manic and depressive psychosis in bipolar disorder. *Transl. Psychiatry*. **8**, 188 (2018). 10.1038/s41398-018-0242-3 PMC6131184 30201969

[47] Pain O (2018). Genome-wide analysis of adolescent psychotic-like experiences shows genetic overlap with psychiatric disorders. Am. J. Med Genet. B Neuropsychiatr. Genet..

[48] Pasman JA (2018). GWAS of lifetime cannabis use reveals new risk loci, genetic overlap with psychiatric traits, and a causal influence of schizophrenia. Nat. Neurosci..

[49] Baron-Cohen S (2006). The hyper-systemizing, assortative mating theory of autism. Prog. Neuropsychopharmacol. Biol. Psychiatry.

[50] Parellada, M. What does the future hold for Asperger syndrome. *Rev Psiquiatr Salud Ment* (*accepted for publication*). 10.1016/j.rpsm.2019.05.002. 10.1016/j.rpsm.2019.05.002 31248755

[51] Duffy FH, Shankardass A, McAnulty GB, Als H (2013). The relationship of Asperger’s syndrome to autism: a preliminary EEG coherence study. BMC Med..

[52] Enticott PG, Bradshaw JL, Iansek R, Tonge BJ, Rinehart NJ (2009). Electrophysiological signs of supplementary-motor-area deficits in high-functioning autism but not Asperger syndrome: an examination of internally cued movement-related potentials. Dev. Med. Child. Neurol..

[53] Luckhardt C, Jarczok TA, Bender S (2014). Elucidating the neurophysiological underpinnings of autism spectrum disorder: new developments. J. Neural Transm. (Vienna)..

[54] Barahona-Corrêa JB, Filipe CN (2015). A Concise history of Asperger syndrome: the short reign of a troublesome diagnosis. Front. Psychol..

[55] Jeste SS, Geschwind DH (2014). Disentangling the heterogeneity of autism spectrum disorder through genetic findings. Nat. Rev. Neurol..

[56] Jansen AG (2020). Psychiatric polygenic risk scores as predictor for attention deficit/hyperactivity disorder and autism spectrum disorder in a clinical child and adolescent sample. Behav. Genet..

[57] Mazzone L, Ruta L, Reale L (2012). Psychiatric comorbidities in asperger syndrome and high functioning autism: diagnostic challenges. Ann. Gen. Psychiatry.

