# Supplementary material for: Psychiatric comorbidities in Asperger syndrome are related with polygenic overlap and differ from other Autism subtypes
Source: Transl Psychiatry. 2020 Jul 30;10:258. doi: 10.1038/s41398-020-00939-7 (PMC7393162; doi:10.1038/s41398-020-00939-7)

**Supplementary figure 1**. Correlation between ASD and comorbid disorders tested in the current study (SCZ, MDD, ADHD, ANX and OCD) at the pTDT deviation level. ▪ P < 0.1; * P < 0.01; ** P < 0.001; *** P < 0.0001. Spearman correlation values, plot representation and pTDT distribution are represented above, below and within the diagonal line, respectively.


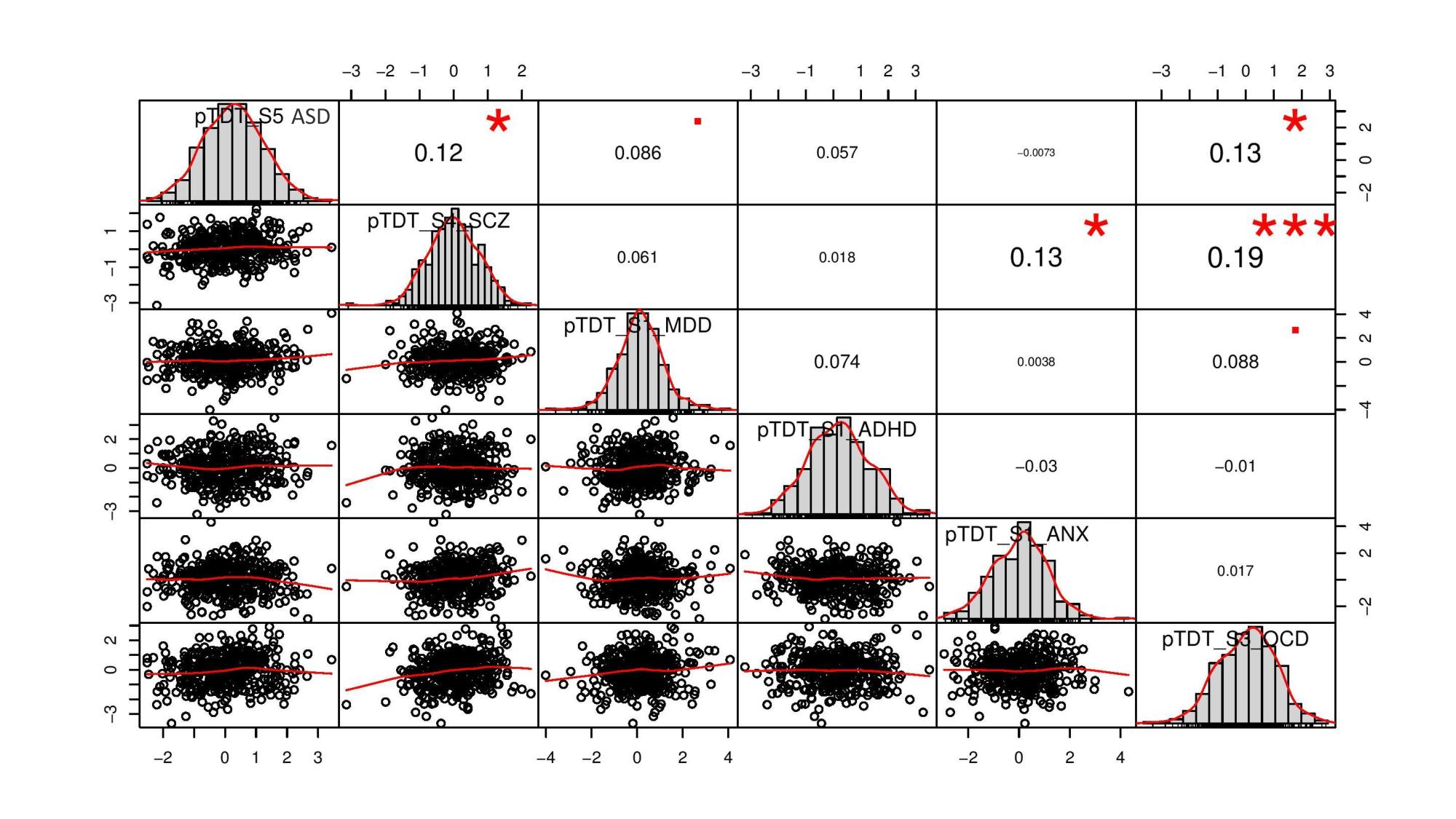


**Supplementary figure 2**. Dendogram resulting from hierarchical clustering procedure. 2 main clusters are described (C1 in yellow, C2 in blue). Detailed procedure is fully described in **methods**. Cluster classification and sample IDs are described in **supplementary table 4**.


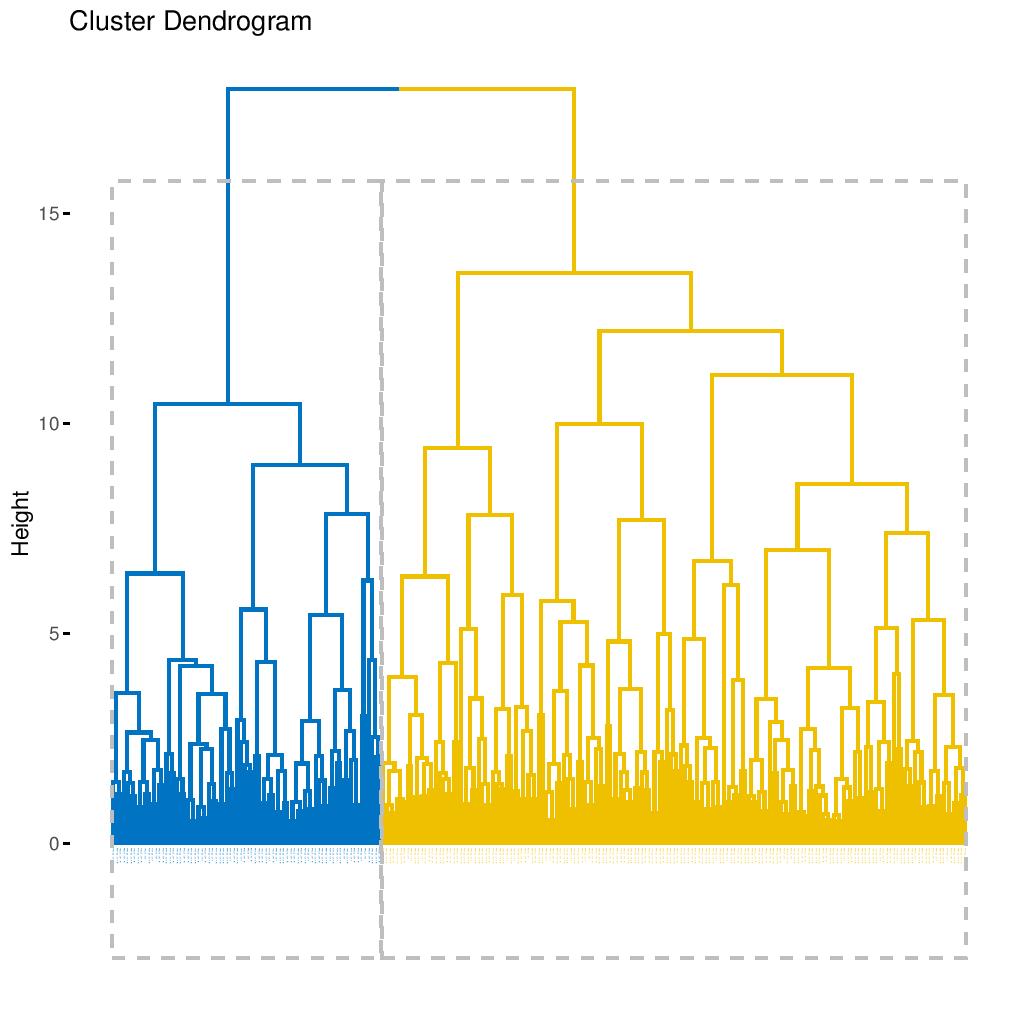


**Supplementary figure 3**. Cluster silhouette plot performed to validate hierarchical clustering method. Samples with negative silhouette width values are described in **supplementary table 4**.


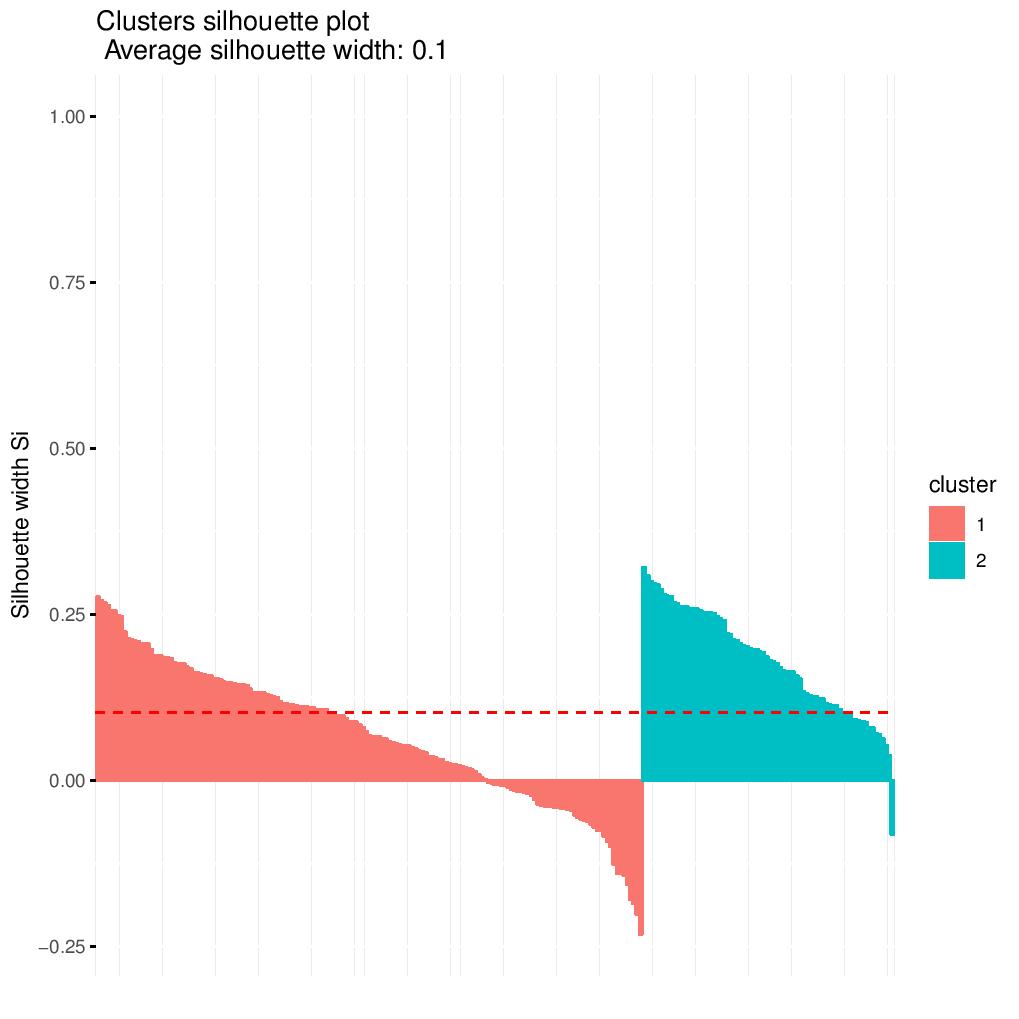

Supplement: Supplementary file 1 — Supplementary Information [file 41398_2020_939_MOESM1_ESM.doc]
